# Molecular puzzle of insulin: structural assembly pathways and their role in diabetes

**DOI:** 10.3389/fcell.2025.1502469

**Published:** 2025-02-20

**Authors:** Edyta Urbaniak, Sara Henry, Maciej Lalowski, Malgorzata Borowiak

**Affiliations:** 1 Institute of Molecular Biology and Biotechnology, Adam Mickiewicz University, Poznan, Poland; 2 Meilahti Clinical Proteomics Core Facility, HiLIFE, University of Helsinki, Helsinki, Finland; 3 Center for Cell and Gene Therapy, Stem Cell and Regenerative Medicine Center, Baylor College of Medicine, Texas Children’s Hospital, Methodist Hospital, Houston, TX, United States; 4 McNair Medical Institute, Baylor College of Medicine, Houston, TX, United States

**Keywords:** UPR, ER stress, insulin, processing, folding, FKBP2, ER, Golgi network

## Abstract

Properly folded proteins are essential for virtually all cellular processes including enzyme catalysis, signal transduction, and structural support. The cells have evolved intricate mechanisms of control, such as the assistance of chaperones and proteostasis networks, to ensure that proteins mature and fold correctly and maintain their functional conformations. Here, we review the mechanisms governing the folding of key hormonal regulators or glucose homeostasis. The insulin synthesis in pancreatic β-cells begins with preproinsulin production. During translation, the insulin precursor involves components of the endoplasmic reticulum (ER) translocation machinery, which are essential for proper orientation, translocation, and cleavage of the signal peptide of preproinsulin. These steps are critical to initiate the correct folding of proinsulin. Proinsulin foldability is optimized in the ER, an environment evolved to support the folding process and the formation of disulfide bonds while minimizing misfolding. This environment is intricately linked to ER stress response pathways, which have both beneficial and potentially harmful effects on pancreatic β-cells. Proinsulin misfolding can result from excessive biosynthetic ER load, proinsulin gene mutations, or genetic predispositions affecting the ER folding environment. Misfolded proinsulin leads to deficient insulin production and contributes to diabetes pathogenesis. Understanding the mechanisms of protein folding is critical for addressing diabetes and other protein misfolding-related diseases.

## 1 Introduction

Protein folding is a fundamental process in cellular biology, in which proteins adopt specific three-dimensional structures essential for their function. The linear sequence of amino acids, known as the primary structure, inherently encodes the information required for the protein to attain its correct three-dimensional conformation. The folding process is driven by various weak interactions, including hydrogen bonds, hydrophobic interactions, electrostatic interactions, and van der Waals forces. These interactions guide the polypeptide chain toward its most energetically favorable conformation, the native state. Properly folded proteins are necessary for nearly all cellular processes, including enzyme catalysis, signal transduction, and providing structural support. Under normal conditions, the predominant protein structure observed in cells is the native conformation. For many proteins, the most prominent structural motif of the functional protein in its native conformation is a right-handed spiral coil, known as the α-helix or properly ordered β-sheet, which allows the protein to perform its proper biological functions (Dobson, 2003). In toxic proteins, on the other hand, this process is disrupted, resulting in abnormal folding and the appearance of a “wrong” conformation. In this case, the protein becomes “saturated” with β-sheets, leading to its rigid, more stable, and more cross-linked structure. This incorrect structure is resistant to degradation, often resulting in the formation of amyloid aggregates. These aggregates have a characteristic organized structure consisting of β-sheets arranged in parallel or antiparallel, leading to the formation of stable amyloid fibrils (Dobson, 2003).

### 1.1 Insulin folding and diabetes

The primary focus of this review is to elucidate the crucial mechanisms governing insulin (INS, human UniProt/Swiss-Prot identifier, ID: P01308) maturation, particularly INS folding predominantly occurring within the endoplasmic reticulum (ER) of pancreatic β-cells. INS plays an essential role in glucose metabolism and homeostasis. Its functionality is intricately linked to proper folding and structural assembly, which involve the formation of specific disulfide bonds that stabilize the interaction between the A and B chains of the mature INS molecule (Lee et al., 2021; Liu et al., 2014; Mayer et al., 2007). We will explore the critical role of molecular chaperones and folding catalysts in assisting proinsulin (PROINS), the INS precursor, in attaining its native conformation. These proteins, such as members of the protein disulfide isomerase (PDI) family and the heat shock protein (HSP) family, facilitate the correct formation of disulfide bonds, prevent the formation of misfolded intermediates, and ensure efficient protein trafficking within the secretory pathway.

Misfolding of INS can have detrimental consequences, leading to a diminished pool of functional INS, accumulation of misfolded INS within the ER, and subsequent ER stress implicated in β-cell apoptosis and the development of diabetes (Fonseca et al., 2011; Yong et al., 2021). By comprehensively investigating the molecular mechanisms underlying INS folding and the impact of protein misfolding on β-cell function, this review aims to provide valuable insights into the pathophysiology of diabetes and to identify potential therapeutic targets for the treatment of this prevalent disease.

**FIGURE 1:**
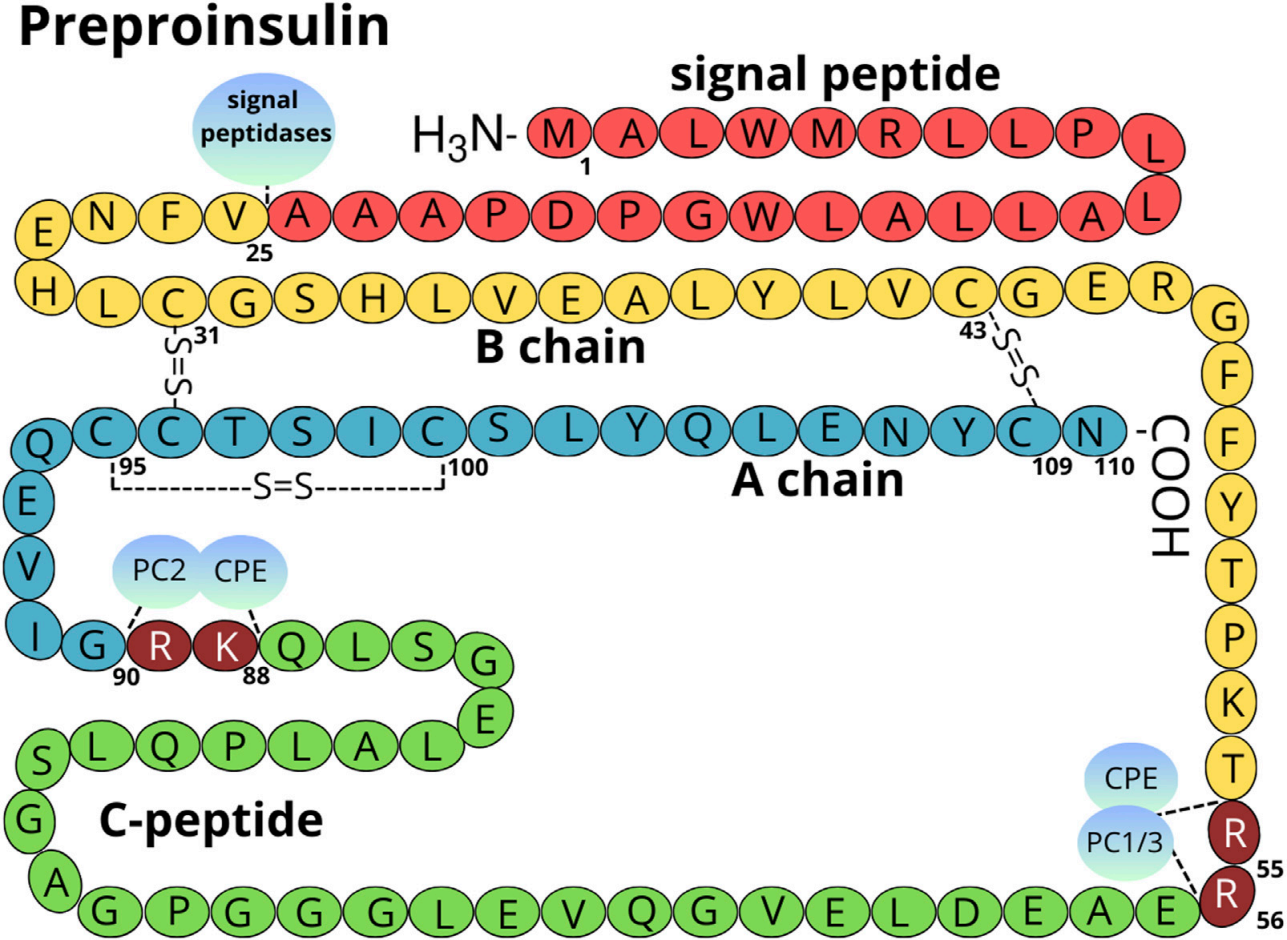
Structure of the preproinsulin molecule. The signal peptide is marked in red, the B chain in yellow, the A chain in light blue, and the C-peptide in green. Amino acids cleaved by PC1/3 (P29120), PC2 (P16519), and CPE (P16870) are marked in maroon (numbering Arg 55-56 and Lys 88-Arg 89 refers to preproinsulin molecule) (Liu et al., 2018). The localization of intra and inter-disulfide bonds is shown. The amino acid numbering is done according to the human preproinsulin canonical sequence (P01308-1).

The pancreas is a glandular organ comprising of two distinct compartments: the endocrine compartment consists of hormone-secreting islets of Langerhans, and the exocrine compartment, composed of ducts and acinar cells that aid digestion. Within the endocrine islets, β-cells are essential for maintaining glucose homeostasis as the sole producers of INS. The β-cells exhibit remarkable efficiency in INS synthesis, with each cell producing up to 6,000 preproinsulin molecules per second (Liu et al., 2018; Schuit et al., 1991). Preproinsulin, the initial precursor, comprises of the A and B chains of mature INS, C-peptide (C-PEP), and a signal sequence. Early in maturation, the signal sequence is first cleaved during translocation into the ER (Figure 1, detailed in chapter 2, Insulin Folding). The resulting PROINS undergoes folding and further processing within the rough ER, where crucial disulfide bonds link A and B chains to form INS (Liu et al., 2018). PROINS biosynthesis constitutes a significant metabolic burden, accounting for 30%–50% of total protein synthesis in β-cells. This energy-intensive process evokes considerable pressure on the β-cell secretory pathway, especially the ER, and significantly impacts PROINS folding (Sun et al., 2015). Notably a substantial proportion of preproinsulin molecules misfolds (approximately 30%) under physiological conditions, which can be further exacerbated by ER stress and increased INS demand (Sun et al., 2015).

Eukaryotic cells have developed intricate mechanisms, including molecular chaperones and proteostasis networks, to ensure proper protein folding and maintain protein functional conformations (Saibil, 2013). To counteract/mitigate the detrimental effects of increased ER stress and adapt to fluctuating INS secretion demands, β-cells have evolved well-regulated mechanisms for PROINS folding (Fonseca et al., 2011; Yong et al., 2021). Molecular chaperones are crucial for proper proinsulin folding by facilitating disulfide bond formation and eliminating improperly folded proteins, thereby maintaining β-cell homeostasis (Saibil, 2013).

### 1.2 The role of genetic variants in insulin misfolding

Genetic variants, particularly mutations in the *INS* gene and genes coding for folding-associated proteins, can predispose individuals to INS misfolding and diabetes (for an overview see Supplementary Table S1). The incorrectly folded PROINS, resulting from these genetic alternations, has been associated with impaired INS production and the onset of diabetes, including mutant *INS*-gene-induced diabetes of Youth (MIDY) and type 2 diabetes (T2D). In MIDY, specific mutations in the *INS* gene induce structural abnormalities that increase the protein’s tendency/propensity to misfold and form insoluble aggregates in the ER (Arunagiri et al., 2018). Examination of seven MIDY mutants, namely G(B8)V, Y(B26)C, L(A16)P, H (B5)D, V(B18)A, R(Cpep +2)C, E(A4)K, demonstrated that six of them [except V(B18)A] completely blocked in export from the ER in pancreatic β-cells (Haataja et al., 2021). Moreover, variants in genes related to ER stress response including *WFS1*, *HSPA5*, *EIF2AK3*, *DNAJC3* [Supplementary Table S1 and (Arunagiri et al., 2018)] or protein transport, i.e., *GLUT2* (Mueckler et al., 1994) or solute carriers protein family (i.e., *SLC16A1*/MCT11, *SLC16A13*/MCT13, *SLC25A7*/UCP1, *SLC25A8*/UCP2, *SLC25A9*/UCP3, *SLC30A8*/ZnT8; reviewed in Mueckler et al. (1994) can further burden β-cell secretion systems, accelerating disease progression and worsening INS deficiency. The significance of protein folding extends beyond disease prevention. These mechanisms are vital for cell survival and function, emphasizing the importance of protein folding in both health and disease states (Diaz-Villanueva et al., 2015). Understanding protein folding and its impact on cellular function is crucial for developing new therapeutic strategies and improving existing treatments for diabetes and other diseases (Mukherjee et al., 2015).

## 2 Insulin folding: a key step in biosynthesis

**FIGURE 2:**
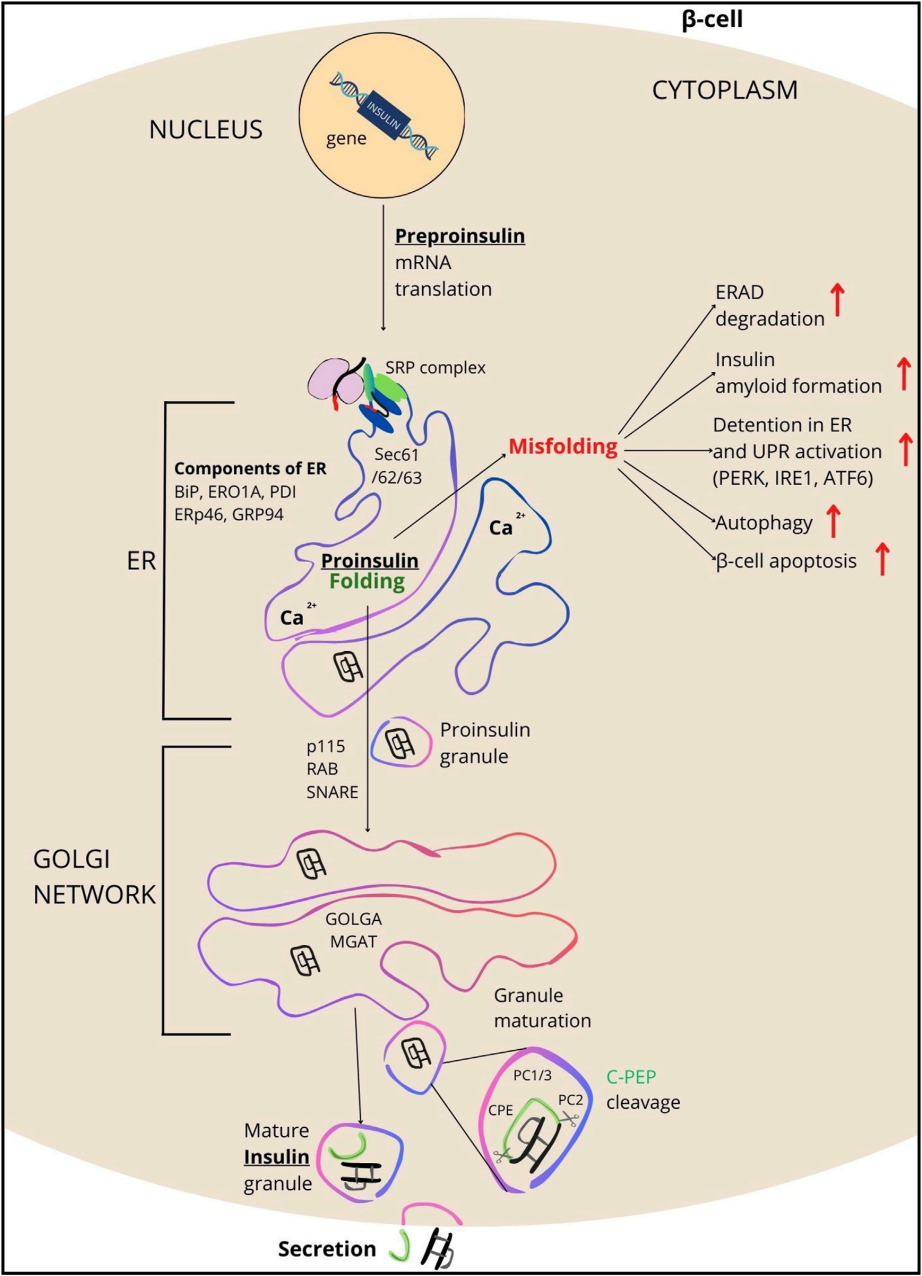
Overview of INS processing. INS synthesis begins with the formation of preproinsulin, a 110-amino acid precursor. Guided by the signal peptide, preproinsulin enters the endoplasmic reticulum (ER), where it is cleaved by signal peptidase, transitioning the polypeptide into proinsulin (PROINS). This conversion is facilitated by various ER components including SRP, SEC61/62/63 complex proteins, BIP, ERO1A, PDI, TXNDC5 (ERp46/Q8NBS9), GRP94, and the ERAD protein complex (which removes misfolded proteins). PDI catalyzes the formation of three disulfide bonds in the ER for proper PROINS folding. In the next step, PROINS traverses through the Golgi network, where the post-translational processing of INS occurs. It involves posttranslational modifications to PROINS, including glycosylations, sorting, and packaging into vesicles that form secretory granules (SG) upon dissociation from the trans-Golgi. Proteins such as p115 (USO1/O60763), RAB/Q6IQ22, SNARE/O95249, GOLGA/Q08379, and MGAT/Q09327 participate in these processes. Within SG, enzymes such as PC1/3, PC2, and CPE cleave the C-PEP from PROINS (for details see Supplementary Table S2), resulting in the production of mature INS, which subsequently is secreted outside the β-cells.

INS folding mainly takes place in the ER, where it gains the tertiary structure, essential for pursuing its biological function (Lee and Lee, 2022; Liu et al., 2014; Mayer et al., 2007). The ER provides an optimal milieu for the folding process, such as the suitable pH and the presence of accessory proteins and cleaving enzymes. Inside ER, INS goes through a series of biochemical reactions (Braakman and Hebert, 2013) involving various proteins, including protein chaperones and disulfide isomerases. Protein chaperones including HSP70 (P0DMV8 and P0DMV9) and GRP78/BiP (immunoglobulin-binding protein/P11021) provide stability and a suitable framework for proper alignment of INS polypeptide chains. Disulfide isomerases, such as protein disulfide isomerase (PDI/P07237) catalyze the formation of disulfide bridges between cysteine (Cys) residues in proteins. Regarding INS, disulfide isomerases facilitate the formation of disulfide bridges between Cys residues in the A and B chains, a crucial step for achieving the correctly folded structure of the mature polypeptide (Mayer et al., 2000). The interplay between chaperones and disulfide isomerases is essential for the efficient folding of INS in the ER. These proteins not only facilitate the folding process but also act as quality control mechanisms, preventing the accumulation of misfolded proteins and activating signaling pathways responsible for the stress response when the folding process is disrupted. Therefore, ER-mediated INS processing is a key step in the biosynthesis of the hormone (Figure 2) (Liu et al., 2014; Saibil, 2013).

Key stages of INS folding encompass: a) Preproinsulin synthesis and translocation to the ER, b) Oxidative folding and disulfide bond formation, chaperone-mediate folding, and prolyl isomerization, c) Glycosylation and further stabilization, d) Final disulfide bond formation and transport.

### 2.1 The journey of insulin folding

#### 2.1.1 Preproinsulin synthesis and translocation to the ER

**FIGURE 3:**
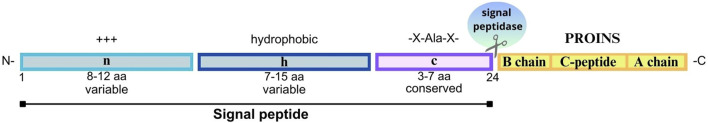
Structure of the signal peptide molecule and PROINS. The signal peptide consists of three key regions: N-region (*n*)- a variable, positively charged region (+++) at the beginning of a signal peptide that helps to direct preproinsulin to endoplasmic reticulum (ER); H-region (*h*) - a hydrophobic region that facilitates the binding of a signal peptide to the ER membrane, and C-region (*c*) - a conserved region recognized and cleaved by signal endopeptidase (SP). The most commonly recognized motif is -X-Ala-X- (where X represents any amino acid), in which alanine serves as a key amino acid at the cleavage site. The length of each segment is indicated below (in amino acids, aa). The PROINS part is marked in yellow, built of the B chain, C-peptide, and A chain, respectively. The human signal peptide molecules consist of 24 aa (numbering according to human preproinsulin sequence P01308-1).

INS synthesis commences with the translation of a single-chain preproinsulin mRNA on ribosomes attached to the ER membrane. Structural analysis of preproinsulin reveals an N-terminus signal peptide, followed by the A chain, C-PEP, and B chain (Figures 1, 3) (Liu et al., 2014). The signal peptide composed of 24 amino acids in humans, is divided into three functional regions: *n*, *c*, and *h*, whose lengths can vary across species (in human: *n* = 3, *h* = 14, *c* = 7) (Gutierrez Guarnizo et al., 2023). The highly conserved *c* region is recognized by endopeptidase-signal peptidase, while the *h* and *n* regions show high variability (Figure 3) (Hiss and Schneider, 2009; von Heijne, 1990). The primary function of the signal peptide is to interact with the signal recognition particle (SRP) and the Sec62 homolog, preproinsulin translocation factor (SEC62/Q99442; Supplementary Table S2) (Liu et al., 2021), facilitating the binding of preproinsulin to receptors on the ER surface, and its subsequent translocation across the membrane (Liu et al., 2014). Upon translocation across the ER membrane, signal peptidase located on the inner side of the ER membrane cleaves the preproinsulin. This cleavage generates PROINS and releases a separate signal peptide for subsequent degradation.

#### 2.1.2 Oxidative folding and disulfide bond formation

**FIGURE 4:**
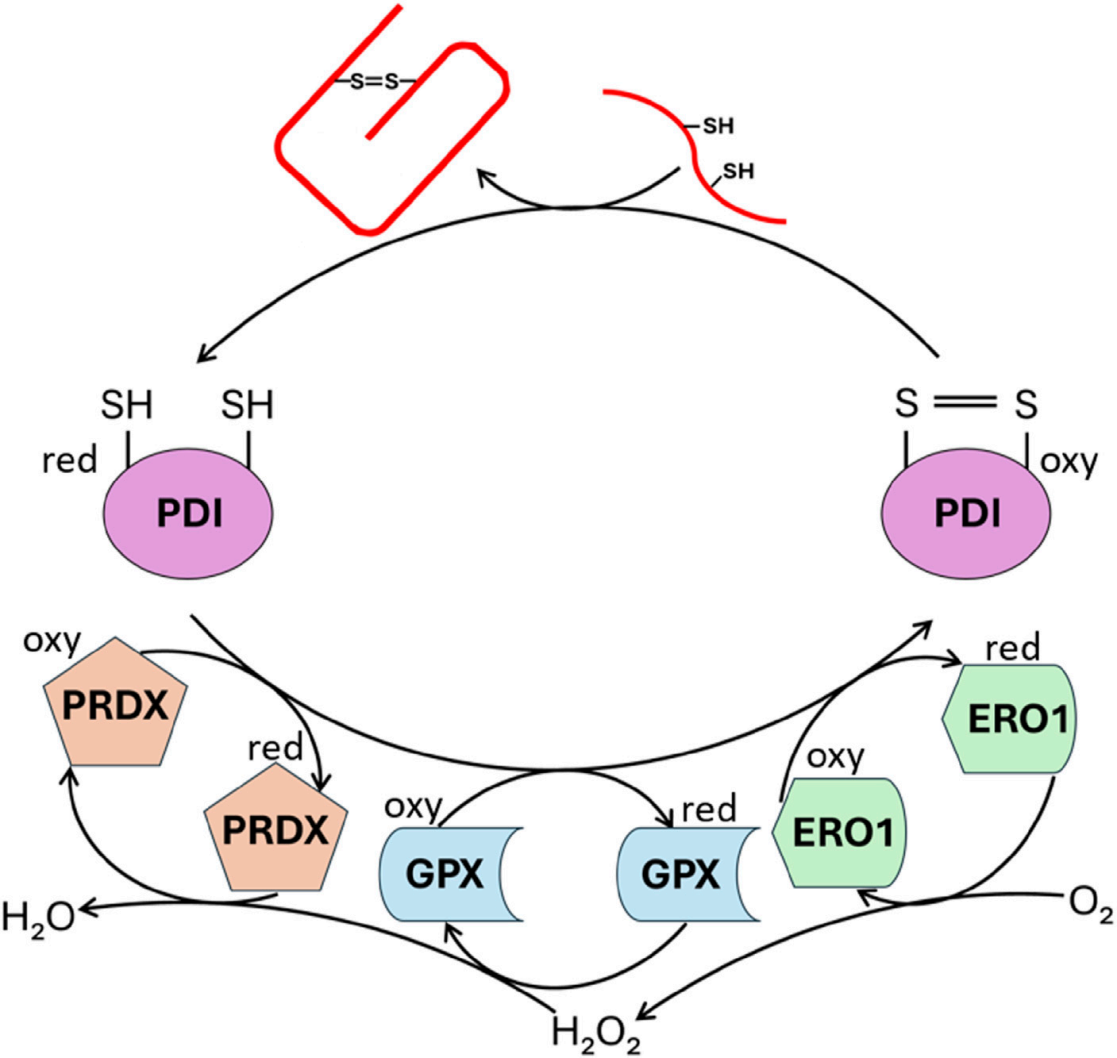
Enzymatic catalysis of PROINS disulfide bond formation. The figure illustrates the action of enzymes involved in establishing PROINS’s disulfide bonds. Protein disulfide isomerase (PDI) catalyzes the formation of these bonds, while enzymes like peroxiredoxin (PRDX), glutathione peroxidase (GPX), and ERO1 oxidize PDI, enabling it to continue its function. The reduction of molecular oxygen is coupled to these oxidation reactions. “Oxy” denotes the oxidized form, and “red” represents the reduced form. The unfolded PROINS is depicted as a stretched red line structure with attached SH groups. The structure connected by the S=S bridge represents the correctly folded PROINS.

Within ER, PROINS undergoes a series of conformational changes to attain its native structure. The ER lumen oxidative status and the concerted action of chaperone proteins promote the formation of intra- and intermolecular disulfide bonds between specific cysteine residues at 31–96, 43–109, and 95–100 sites (numbering using canonical human preproinsulin sequence P01308-1, Figure 1). These three disulfide bridges stabilize the protein three-dimensional structure aiding in proper INS folding (Liu et al., 2014; Mayer et al., 2007). PDI and other members of the PDI family catalyze the formation and rearrangement of these disulfide bonds, ensuring correct pairing. In its oxidized form, PDI possesses a specific disulfide bond (Cys-X-X-Cys) within its active site, which is transferred to the substrate (PROINS) during the catalysis process, resulting in the reduction of PDI. To regain its catalytic activity PDI must be re-oxidized by the ER-localized oxireductin 1 (ERO1/Q96HE7), which acts as the ultimate electron acceptor in the process. ERO1 catalyzes the conversion of oxygen (O_2_) to hydrogen peroxide (H_2_O_2_), generating the reactive oxygen species (ROS) as a byproduct (Araki and Nagata, 2011; Liu et al., 2018). The accumulation of ROS within the ER can be detrimental and necessitates detoxification. Peroxiredoxin 4 (PRDX4/Q13162), another ER-resident enzyme, removes H_2_O_2_ and contributes to the oxidation of PDI family proteins, thus indirectly facilitating disulfide bond formation. Through the interplay of these two mechanisms, two disulfide bonds are generated from a single molecule of reduced oxygen. Furthermore, other H_2_O_2_-reducing enzymes in the ER, such as glutathione peroxidase (GPX) superfamily, particularly GPX7 (Q96SL4) and GPX8 (Q8TED1), play crucial roles in maintaining a balanced redox environment and supporting the oxidative activity of PDI family proteins (Figure 4) (Bulleid and Ellgaard, 2011; Kanemura et al., 2020).

#### 2.1.3 Chaperone-mediated folding

The nascent PROINS chain, upon its synthesis on ribosomes, encounters a complex network of molecular chaperones within the ER. These chaperone proteins, including HSP70, BiP/GRP78, GRP94 (P14625), and HSP90 (P07900) play an important role in stabilizing the nascent polypeptide and guiding its folding process (Supplementary Table S2). These chaperones recognize and bind to exposed hydrophobic regions on the nascent PROINS chain preventing aggregation and subsequent degradation. Furthermore, chaperones help to maintain the nascent polypeptide in a partially unfolded state, allowing it to explore different conformations and reach the most energetically favorable native structure. Lastly, chaperones actively guide the folding process by interacting with specific regions of the PROINS molecule and promoting the formation of crucial secondary and tertiary structures.

#### 2.1.4 Prolyl isomerization

Proline residues within PROINS sequence present a unique challenge for protein folding. Proline, due to its cyclic structure, can adopt *cis* or *trans* conformations. The peptide bond preceding proline (the X-Pro bond) can rotate between these two conformations, significantly impacting the overall protein structure and folding kinetics pathways. The slow spontaneous interconversion between *cis* and *trans* proline conformations can trap the folding process, preventing the protein from reaching its native state. In proline, only one conformation, usually *trans,* is favorable. Proteins involved in the correct folding of PROINS may already be active before disulfide bond formation (Hoefner et al., 2023; Jang et al., 2019). One such protein is FK506 Binding Protein 2 (FKBP2/P26885), which catalyzes the *cis*/*trans* isomerization of X-pro bonds (Hoefner et al., 2023; Milovic and Kostic, 2003). By accelerating this rate-limiting step, FKBP2 facilitates efficient protein folding and helps to overcome potential kinetic traps. Taking into account the favorable *trans* conformation of proline, one can predict that half of the PROINS will receive *trans*-proline and fold quickly. The other half will incorporate *cis*-proline and would require FKBP2 to speed up its folding. Consequently, FKBP2 activity can be viewed as the gatekeeper for half of the produced PROINS. Interestingly, FKBP2 expression is induced in response to the buildup of unfolded proteins during ER stress (Ostrovsky et al., 2009). Furthermore, recent studies using single-cell RNA-sequencing of human primary β-cells, showed the decreased *FKBP2* expression in β-cells from T2D donors (Hoefner et al., 2023; Milovic and Kostic, 2003).

#### 2.1.5 Glycosylation and further stabilization

Post-translational modifications (PTMs) in the Golgi apparatus are essential for the proper maturation of INS granules in pancreatic β-cells, significantly influencing INS biosynthesis and secretion (Omar-Hmeadi and Idevall-Hagren, 2021). During its transit through the Golgi, PROINS experiences various PTMs, including glycosylation, which adds carbohydrate moieties that enhance protein stability and solubility (Yang et al., 2023). Following glycosylation, Calnexin (CANX/P27824) and Calreticulin (CALR/P27797) lectin chaperones residing in the ER membrane and lumen, respectively, recognize specific glycan structures and bind to the glycosylated PROINS, further stabilizing and promoting INS maturation (Swanton et al., 2003). This interaction also provides a platform for other chaperones and folding enzymes to interact with the PROINS molecule. Glycosylation serves also as a crucial quality control mechanism. If the protein folds incorrectly, the glycan structure may be modified, leading to the release of protein from the lectin chaperone complex and potentially targeting it for degradation.

#### 2.1.6 Final disulfide bond formation and transport to Golgi apparatus

The formation of a disulfide bond in PROINS is a complex, tightly regulated process that ensures the stability and functionality of the INS precursor. PDI plays a central role in catalyzing the formation and rearrangement of these disulfide bonds. ERp57/PDIA3 (P30101), another member of the PDI family, supports PDI in the final stages of disulfide bond formation, ensuring the correct pairing of cysteine residues (Yi et al., 2018).

#### 2.1.7 Prohormone processing and packaging

Within the Golgi, PROINS undergoes a series of proteolytic cleavages.

1) PC1/3 Cleavage: Proprotein convertase 1/3 (PC1/3) cleaves PROINS at the B chain and C-PEP junction, removing a short peptide sequence,
2) CPE Cleavage: Carboxypeptidase E (CPE) removes dibasic amino acids (Arg-Arg) from the cleavage sites generated by PC1/3, resulting in the formation of INS and C-PEP,
3) Packaging: Mature INS is then packaged into secretory vesicles within the Golgi. These vesicles also contain zinc (Zn^2+^) and calcium (Ca^2+^) ions, which play crucial roles in the formation of INS hexamers, the stable storage form of INS within the β-cells.

Once properly folded, coiled PROINS forms dimers and is transported from the ER to the Golgi apparatus (Amirruddin et al., 2021; Liu et al., 2014). This transport involves the formation of specialized vesicles. Vesicle formation initiates with the activation of the GTPases Sar1 (SAR1A/Q9NR31 and SAR1B/Q9Y6B6). Activated Sar1 recruits the Sec23 (SEC23A/Q15436; SEC23B/Q15437) and Sec24 family proteins (SEC24A/O95486) to the ER membrane. Sec23 and Sec24, along with other coat proteins like Sec13 (SEC13/P55735) and Sec31(SEC31/O94979), assemble into a cage-like structure known as the COPII coat (Saito et al., 2017). Sec13 and Sec31 proteins form the outer layer of the vesicle, enabling its scission from the ER membrane (Gomez-Navarro and Miller, 2016). This coat drives the budding of the vesicle from the ER membrane, encapsulating PROINS and other cargo proteins. The Sec24 subunit of the Sec23/Sec24 complex plays a crucial role in cargo selection, ensuring that specific proteins, including PROINS, are efficiently incorporated into the forming vesicles.

The loaded vesicles travel to the Golgi membrane, where they fuse with the Golgi membranes and release their protein cargo into the Golgi lumen. Within the Golgi, PROINS undergoes various processing stages, beginning with the removal of mannose residues by mannosidase I (MAN1A1/P33908; MAN1A2/O60476; MAN1A3/Q9NR34). Subsequently, galactose residues are added by galactosyltransferase (GalT, group of galactose-1-phosphate uridylyltransferase (GALT) proteins, e.g., P15291), and N-acetylglucosamine residues are attached by N-acetylglucosaminyltransferase (GlcNAcT, group of MGAT proteins, e.g., Q09327), contributing to proper protein folding. The resulting glycoproteins are then packed into the secretory vesicles that bud off from the Golgi apparatus (Fisher and Ungar, 2016; Nagae et al., 2020). Within immature vesicles, the PROINS molecule undergoes a series of proteolytic cleavages. First proprotein convertase 1/3 (PC1/3) cleaves PROINS at the B chain and C-PEP junction, specifically at residues 32–33. This cleavage enables the subsequent removal of additional amino acids (Arg31-Arg32) by carboxypeptidase E (CPE) (Figure 2). Next, PC2 cleaves PROINS at the C-PEP-B chain junction, where two basic amino acids (Lys64-Arg65) are present, which are subsequently cleaved by CPE (Figure 2), resulting in the formation of INS and C-PEP (Furuta et al., 1998; Liu et al., 2014). In the Golgi apparatus, mature INS is stored in secretory vesicles containing Zn^2+^ and Ca^2+^ ions, which facilitate the formation of an inactive, INS hexamers, the stable storage form of INS within the β-cells. To regain its activity, INS undergoes a monomerization, dissociating from a hexamer into a monomeric form capable of interacting with INS receptors (Dunn, 2005). Monomerization is crucial for efficient blood glucose regulation, enabling INS to react rapidly with receptors on target cells.

## 3 INS secretion and action

INS secretion from pancreatic β-cells is a tightly regulated process crucial for maintaining glucose homeostasis, ensuring that blood glucose levels remain within a narrow range. This dynamic process involves a complex interplay of glucose metabolism, ion channel activity, and intracellular signaling pathways. Glucose-stimulated INS secretion occurs in two distinct phases, initiated by glucose entering β-cells via glucose transporter 2 (GLUT2/P11168) (Leturque et al., 2005). Glucose is then converted to glucose-6-phosphate by glucokinase through glycolysis, followed by adenosine triphosphate (ATP) generation through cellular respiration (Barg et al., 2002; Jitrapakdee et al., 2010). The increase in intracellular ATP leads to the closure of ATP-sensitive potassium channels, leading to elevated intracellular K⁺ concentration and membrane depolarization. Consequently, L-type, voltage-gated, calcium channels open, allowing Ca^2+^ ions to enter the cell, triggering the rapid and transient release of pre-synthesized INS stored in readily releasable pools near the plasma membrane via exocytosis. This corresponds to the first phase of INS secretion (Barg et al., 2002).

The second phase of INS secretion is slower and more sustained, involving the mobilization of INS granules from reserve pools deeper within the cell to the plasma membrane for subsequent exocytosis. This sustained release ensures a continuous supply of INS to maintain glucose homeostasis over a longer period.

While glucose is the primary stimulus, various other factors influence INS secretion, including neural signals (parasympathetic stimulation enhances INS release, whereas sympathetic stimulation inhibits it), and hormones like incretins, glucagon-like peptide-1 (GLP-1), and gastric inhibitory polypeptide (GIP), which potently enhance glucose-stimulated INS secretion (Hyun and Sohn, 2022; Santos-Hernandez et al., 2024). Hormones such as glucagon and adrenaline, realized primarily in response to hypoglycemia, decrease INS secretion and counter the effects of INS by stimulating glucose production and release from the liver (Gray et al., 2024; Sluga et al., 2022). Certain amino acids, especially arginine and leucine, can directly stimulate INS secretion independently of glucose (Halperin et al., 2022; Yang et al., 2010). Free fatty acids can also modulate INS secretion, though chronic exposure to elevated levels of free fatty acids can impair β-cell function, contributing to T2D (Ivovic et al., 2024).

Once secreted into the bloodstream, INS binds to its receptors on target tissues such as muscle, liver, and adipose tissue, triggering intracellular signaling cascades that ultimately lead to the glucose uptake. This process involves the translocation of glucose transporter 4 (GLUT4/P14672) from intracellular compartments, such as the trans-Golgi network, to the plasma membrane, allowing for the facilitated diffusion of glucose into cells and thereby lowering blood glucose levels and restoring metabolic balance (Leto and Saltiel, 2012).

INS granules initially form as immature secretory granules within the trans-Golgi network (TGN), where PROINS and other granule components are packaged together. These immature granules are large and loosely packed, containing both PROINS and enzymes necessary for its cleavage into INS and C-PEP (Guest, 2019). Granule maturation involves condensation of contents, driven by acidification of the granule lumen via vacuolar H^+^-ATPase (v-ATPase), which promotes INS crystallization into dense-core structures. This dense packing allows β-cells to store high concentrations of biologically inactive INS until secretion is triggered (Omar-Hmeadi and Idevall-Hagren, 2021).

During maturation, the granules also undergo sorting and removal of byproducts, including C-PEP and cleaved fragments, which are either directed to constitutive secretory pathways or degraded. Misprocessing or accumulation of misfolded proteins can impair granule function and INS secretion (Omar-Hmeadi and Idevall-Hagren, 2021). Molecular chaperones, like chromogranin A, assist in granule biogenesis by promoting condensation and sorting of contents. Mature INS granules are specialized organelles with a dense core surrounded by a lipid bilayer containing key membrane proteins for regulated exocytosis, such as SNARE proteins (syntaxin and synaptobrevin) (Elias et al., 2012). Disruptions in INS granule formation or maturation—caused by mutations, oxidative stress, or other defects—can lead to granule dysfunction, misfolded INS, and impaired secretion, contributing to β-cell stress and diabetes progression (Groegler et al., 2024). These processes underscore the critical role of the Golgi apparatus in INS storage and release, ensuring effective glucose homeostasis.

### 3.1 Consequences of INS misfolding

Proteostasis is the homeostasis of maintaining the proteome by regulating protein synthesis, translocation, post-translational modifications, folding/misfolding processes, and protein degradation or conversion of toxic species into less toxic protein aggregates (Verma et al., 2021). Pancreatic β-cells, responsible for INS production, have a high demand for protein synthesis are particularly susceptible to perturbations in proteostasis. When INS folding is compromised, abnormal polypeptide forms arise, leading to instability, inactivation, or even cellular toxicity. Accumulation of misfolded proteins within the ER can trigger the unfolded protein response (UPR). Key components of UPR signaling are shown as follows.

**FIGURE 5:**
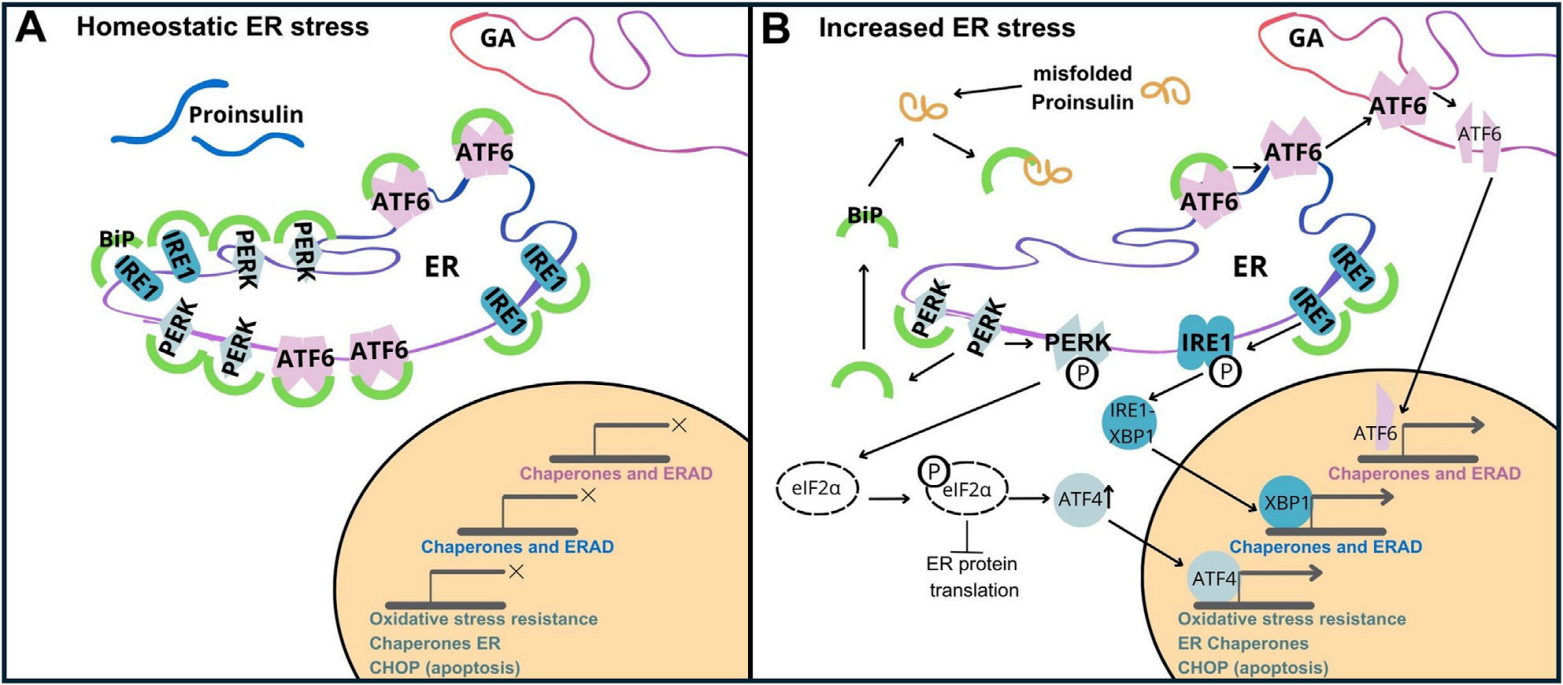
Unfolded Protein Response (UPR) Activation by PROINS Overload. The UPR is mediated by three transmembrane sensor proteins: PERK, IRE1, and ATF6. Under normal conditions (homeostatic ER stress), these proteins are bound to the chaperone protein GRP78/BiP, rendering them inactive **(A)**. During increased ER stress **(B)**, BiP dissociates from sensor proteins to assist in protein folding, initiating the UPR cascade. This leads to dimerization and autophosphorylation of PERK, IRE1, and regulated intramembrane proteolysis of ATF6. Increased PROINS synthesis and accumulation result in elevated BiP binding to PROINS, further contributing to UPR activation. PERK activation triggers the phosphorylation of eIF2α. Phosphorylated eIF2α attenuates global protein synthesis while selectively promoting the translation of activating transcription factor 4 (ATF4). ATF4 upregulates C/EBP homologous protein (CHOP), which is associated with apoptosis.

a) Activating Transcription Factor 6, (ATF6/P18850). ATF6, a transmembrane ER protein, undergoes cleavage in response to unfolded proteins and translocates to the nucleus. In the nucleus, it initiates the expression of genes that increase the ability of the ER to fold (by upregulation of chaperones like BiP/GRP78, and foldases), and removes misfolded proteins by activation of ER-associated degradation (ERAD) pathways. This enables β-cells to adapt to the increased requirements of INS production,
b) Inositol-Requiring Enzyme 1, (IRE1/O75460). IRE1 is another ER transmembrane protein that dimerizes upon activation, and exhibits endoribonuclease activity. In β-cells, activation of IRE1 results in splicing of pre-mRNA X-box Binding Protein 1 (XBP1/P17861), which activates genes responsible for expanding the ER and improving the ability to fold proteins properly. This helps β-cells to cope with the heavy burden of INS synthesis and increases their resistance to ER stress,
c) Protein Kinase RNA-like Endoplasmic Reticulum Kinase (PERK/Q9NZJ5). PERK is an ER transmembrane kinase that, upon activation, phosphorylates the eukaryotic initiation factor 2α (eIF2α), attenuating global protein synthesis and alleviating the influx of new proteins into the ER. In β-cells, this is particularly important because the oversupply of INS or inappropriate folding of INS can lead to the accumulation of abnormal proteins. Additionally, PERK upregulates ATF4 (Cyclic AMP-dependent transcription factor ATF-4/P18848), which enhances the expression of genes associated with stress responses and apoptosis (Figure 5) (Hasnain et al., 2016; Sun et al., 2015).

**FIGURE 6:**
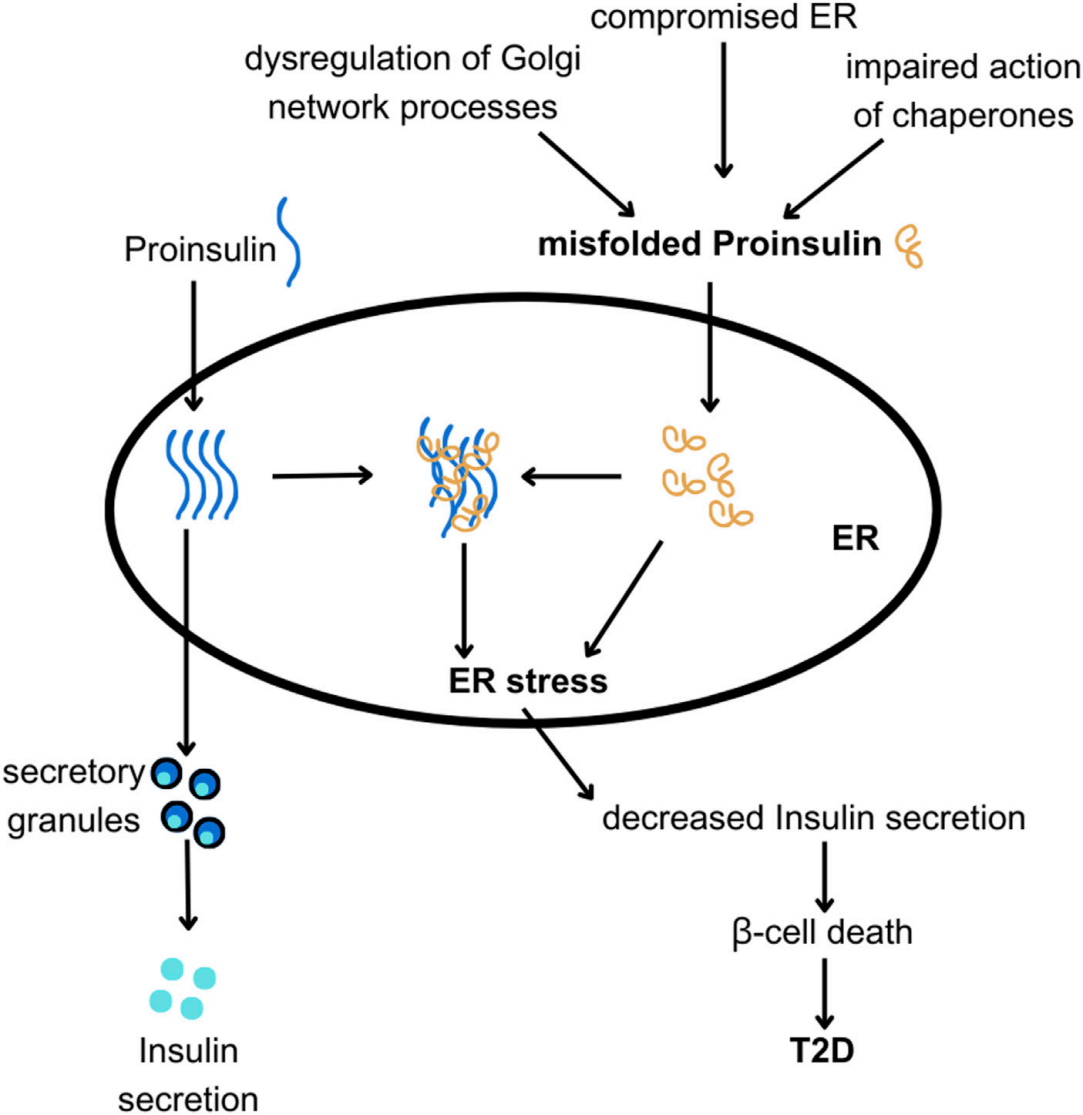
The hypothesis linking ER and Golgi network dysfunction with PROINS misfolding and T2D. PROINS needs to be folded properly by chaperones in the endoplasmic reticulum (ER), to be transported outside of the ER and produce INS. Various factors can lead to PROINS misfolding (as discussed in the text). Misfolded PROINS (shown in orange) can cause other PROINS molecules to become trapped in dysfunctional complexes, impairing their export from the ER. Excessive misfolding of PROINS can also lead to ER stress and subsequent death of β-cells.

When activated, the UPR simultaneously triggers ERAD. ERAD facilitates the removal of misfolded proteins from the ER, targeting them for degradation by proteasomes. While the UPR initially aims to restore ER homeostasis (Araki and Nagata, 2011; Vembar and Brodsky, 2008), prolonged or severe ER stress can overwhelm this compensatory system leading to β-cell dysfunction and death (Fonseca et al., 2011), and ultimately contributing to the pathogenesis of type 1 and type 2 diabetes (Hotamisligil, 2010). Increased synthesis and abundance of newly made PROINS correlates with increased BiP binding to PROINS, associated with dimerization and activation of PERK and IRE1 proteins, and translocation of full-length ATF6 to the Golgi complex for proteolytic processing (Liu et al., 2018). Misfolded PROINS is retained in the ER and targeted for degradation, leading to reduced INS production and secretion (Figure 6) (Marchetti et al., 2007; Rohli et al., 2022). This decreased INS output can contribute to hyperglycemia, which ultimately results in the development of diabetes and other metabolic complications (Sun et al., 2015). Moreover, in some cases, misfolded PROINS can aggregate and form amyloid fibrils, which can interfere with the function of other proteins, damage cellular structures, and further exacerbate β-cell dysfunction. In case of amyloids, the β-sheet structure becomes the dominant secondary form, in which polypeptide chains are arranged in long, parallel, or antiparallel structures, forming rigid and stable aggregates. These β-sheet structures in amyloids are highly organized and denser than in normal proteins, making them resistant to proteolytic degradation. The presence of INS amyloid in pancreatic islets is a pathological hallmark of T2D, contributing to the gradual loss of β-cell mass and cellular dysfunction (Alrouji et al., 2023; Das et al., 2022; Stanciu et al., 2020). In β-cells, the toxic effects of misfolded PROINS can trigger apoptosis, further exacerbating INS deficiency and promoting diabetes progression (Fu et al., 2013; Liu et al., 2021; Zhu et al., 2019). The consequences of abnormal PROINS forms, including the formation of amyloids extend beyond β-cell dysfunction. They can also disrupt mechanisms regulating blood glucose levels and INS response in target tissues, leading to hyperglycemia, INS resistance, and worsened metabolic control in individuals with diabetes (Hodish et al., 2010; Marhfour et al., 2012; Weiss et al., 2000).

Given the critical role of the UPR in healthy β-cell function and development, heightened ER stress or disruptions in associated signaling pathways could contribute, at least in part, to the functional immaturity observed in β-cells derived from human stem cells. Consistent with this hypothesis, *Imeglimin*, an anti-diabetic drug, has been shown to enhance the maturity of stem cell-derived β-cells by improving ER homeostasis and upregulating stress response molecules (Li et al., 2022). Furthermore, deletion of the zinc transporter ZnT8, which is associated with impaired β-cell function, also leads to increased maturity of *in vitro* β-cells, likely through the reduction of ER stress (Ma et al., 2022). These findings suggest that targeting ER stress, either through gene editing or pharmacological interventions, could be a promising strategy to enhance the production of mature, functional β-cells from stem cells. This advancement would bring us closer to having a reliable source of human β-cells that closely mimic the behavior of native primary β-cells.

GWAS studies identified SNPs in folding proteins or ER chaperones in humans with T2D (Cirillo et al., 2018; Rutter, 2016; Watanabe, 2018). This genetic evidence underscores the importance of protein folding in β-cell function. The era of targeted cell therapies in diabetes has begun, with recent successes in reversing preexisting diabetes in mice using gene-edited human stem cell-derived β-cells (Maxwell et al., 2020). Additionally, numerous gene therapy attempts for T2D have demonstrated the feasibility of β-cell-specific cargo delivery and molecular manipulation, offering promising avenues for curing or alleviating T2D symptoms (Ghassemifard et al., 2024; Wal et al., 2024).

Misfolded proteins have been related to illnesses including diabetes, cancer, and neurodegenerative disorders (Diaz-Villanueva et al., 2015; Good and Stoffers, 2020). For the control of glucose levels in diabetics, correct folding of INS and the presence of associated proteins is crucial. Misfolding can affect the synthesis, secretion, and function of INS, which in turn can aggravate diabetes (Eizirik et al., 2008; Liu et al., 2010). Although treatments such as islet transplantation and advancement in β-cell research hold a therapeutic potential, their real-world implementation presents difficulties. Developing more effective therapies for diabetes, and other illnesses requires a thorough understanding of protein folding and its effects on cells (Basu et al., 2023). Improved understanding of the characteristics of protein functional conformations, the conditions in which they operate, and how these cellular defense mechanisms cooperate to preserve protein homeostasis may lead to more effective treatments for various systemic diseases affecting humans.

### 3.2 Therapeutic implications and future research directions

1. PROINS misfolding is linked to deficient INS production and diabetes, as is seen in a variety of contexts: mice with aberrant PROINS-misfolding, human patients with MIDY, animal models, and human patients bearing mutations in critical ER-resident proteins, and in a common variety of T2D (Arunagiri et al., 2018). Understanding the complexities of INS folding and the factors that contribute to misfolding offers opportunities for therapeutic development in diabetes. Boosting the activity of expression of chaperones involved in INS folding could improve INS folding and production and protect β-cells from ER stress. Modulating the redox state or other aspects of the ER environment could potentially enhance INS folding efficiency. Interestingly, recent studies, suggest that chaperones involved in INS folding might play an important role already during β-cells development possibly via modeling the availability of signaling protein and activity of diverse pathways like transforming growth factor beta (TGF-β) (Lee et al., 2021). Further, developing strategies to clear misfolded INS or prevent its aggregation could alleviate ER stress and promote β-cells survival.
2. Research on gene therapies and new drugs: novel possibilities in the treatment of diabetes. Identifying novel therapeutic targets holds the potential for significant improvement in diabetes control (Arnaldi et al., 2012; Breyer, 1995; Tripathy et al., 2015). In the context of INS folding, drug research may focus on substances that facilitate adequate folding of INS during biosynthesis, thereby eliminating the abnormal forms of INS (Chiti and Kelly, 2022). In addition, gene therapies can be used to modify genes encoding proteins involved in the INS folding process, improving pancreatic β-cells function and enhancing INS secretion, i.e., PDI, FKBP2 (Hoefner et al., 2023), or a chaperone Glucose Regulated Protein 94 (GRP94) (Kim et al., 2024).

## 4 Conclusion

Pancreatic β-cells are the only cells in the human body that produce and secrete INS in large quantities. Although both defective INS secretion and impaired INS action in peripheral tissues contribute to T2D, it is now clear that the principal defect lies in the pancreatic β-cells. Mounting evidence points to T2D as a disease with impaired β-cells function rather than reduced β-cells mass. Consequently, to grasp what goes wrong with this process in T2D, we must first understand how INS secretion is regulated physiologically.

Precise regulation of INS secretion is crucial to maintain blood glucose homeostasis. To achieve this, INS secretion has evolved as a multistep process with numerous regulatory mechanisms. The early stages of regulation center on INS production and maturation. A single β-cell can synthesize a substantial quantity of INS, up to 6,000 preproinsulin molecules per second (Liu et al., 2018; Schuit et al., 1991). Consequently, even in the absence of external stimuli, β-cells experience a significant ER stress due to the protein-folding burden associated with PROINS processing. After food intake, INS demand surges dramatically, requiring a substantial increase in PROINS synthesis. This demand further intensifies in INS-resistant states, pregnancy, or obesity. Under normal conditions, approximately 30% of PROINS is misfolded, representing a considerable portion of the total INS pool (Sun et al., 2015). Impaired PROINS folding diminishes INS production and secretion, leading to heightened INS demand. This increased demand exacerbates PROINS misfolding, elevates ER stress, and ultimately contributes to β-cells death. The complex tertiary structure of PROINS, with its three disulfide bonds reliant on the ER redox state, poses challenges to proper folding. High glucose levels further burden β-cells in two ways: first, by stimulating increased PROINS synthesis, and second, by contributing to oxidative and ER stress as part of the diabetic metabolic milieu. The β-cell ER is particularly susceptible to stress due to the substantial protein-folding load imposed by PROINS biosynthesis, which can account for up to 50% of total protein synthesis in stimulated cells. Consequently, hyperglycemia can significantly affect the quality of PROINS folding. Taken together, a major contributor to the development of diabetes-related pathology is the β-cell inability to adequately fold an increasing amount of PROINS, of which the precise mechanisms are not properly understood. Thus, improving our understanding of pancreatic β-cell physiology will potentiate efforts to prevent and treat diabetes. Arguably, the adult human β-cells and their physiological regulation represent the ‟gold standard” for new therapies based on surrogate β-cells, including those derived *in vitro* from stem cells. Recent progress in generating functional INS-producing human cells from stem cells further emphasizes the urgency of elucidating the molecular mechanisms of INS secretion.

## Glossary

ATF4: Activating transcription factor 4
ATF6: Activating transcription factor 6
ATP: Adenosine triphosphate
BiP: Binding immunoglobulin protein
CANX: Calnexin
CALR: Calreticulin
CPE: Carboxypeptidase E
CHOP: CCAT/Enhancer-Binding Protein Homologous Protein
C-PEP: C-peptide
CPOII: Coat Protein Complex II
ERO1: ER Oxidoreductin 1
ER: Endoplasmic Reticulum
ERAD: ER-Associated Degradation
FKBP2: FK506 Binding Protein 2
GALT: Galactose-1-Phosphate Uridylyltransferase
GIP: Gastric Inhibitory Polypeptide
GLcNAcT: Acetylglucosaminyltransferase
GLP-1: Glucagon-Like Peptide-1
GLUT2: Glucose Transporter Type 2
GLUT4: Glucose Transporter Type 4
GPX: Glutathione Peroxidase
GRP78: Glucose-Regulated Protein 78
GRP94: Glucose-Regulated Protein 94
GWAS: Genome-Wide Association Studies
**H**_**2**_ **O**_**2**_: Hydrogen Peroxide
HSP70: Heat Shock Protein 70
HSP90: Heat Shock Protein 90
INS: Insulin
IRE1: Serine/Threonine-Protein Kinase/Endoribonuclease IRE1/Inositol-Requiring Enzyme 1 Alpha
MAN1: Mannosidase I
MIDY: Mutant INS-gene-induced diabetes of Youth
MGAT: Mannosyl (Alpha-1,3-)-Glycoprotein Beta-1,2-N-Acetylglucosaminyltransferase
MODY: Maturity Onset Diabetes of the Young
**O**_**2**_: Oxygen
PC1/3: Proprotein Convertase 1/3
PC2: Proprotein Convertase 2
PDI: Protein Disulfide Isomerase
PDIA/Erp57: Protein Disulfide-Isomerase
PERK: Protein Kinase RNA-Like Endoplasmic Reticulum Kinase/Pancreatic eIF2α Kinase
PRDX4: Peroxiredoxin 4
PROINS: Proinsulin
PTMs: Post-Translational Modifications
ROS: Reactive Oxygen Species
SAR1A: Small COPII Coat GTPase SAR1A
SAR1B: Small COPII Coat GTPase SAR1B
SEC23/24: SEC23 Homolog/SEC24 Homolog
SEC61: Translocation Protein SEC61)
SEC62: Translocation Protein SEC62
SEC63: Translocation Protein SEC63
SG: Secretory Granule
SNARE: Soluble NSF Attachment Protein Receptor
SNP: Single Nucleotide Polymorphism
SP: Signal Peptidase
SRP: Signal Recognition Particle
T2D: Type 2 Diabetes
TGF-β: Transforming Growth Factor beta
TGN: Trans-Golgi Network
TXNDC5: Thioredoxin Domain-Containing Protein 5
UPR: Unfolded Protein Response
vATPase: Vacuolar H^+^-ATPase
XBP1: X-Box Binding Protein 1
