# Correction: Molecular puzzle of insulin: structural assembly pathways and their role in diabetes

**DOI:** 10.3389/fcell.2026.1840980

**Published:** 2026-04-10

**Authors:** Edyta Urbaniak, Sara Henry, Maciej Lalowski, Malgorzata Borowiak

**Affiliations:** 1 Institute of Molecular Biology and Biotechnology, Adam Mickiewicz University, Poznan, Poland; 2 Meilahti Clinical Proteomics Core Facility, HiLIFE, University of Helsinki, Helsinki, Finland; 3 Center for Cell and Gene Therapy, Stem Cell and Regenerative Medicine Center, Baylor College of Medicine, Texas Children’s Hospital, Methodist Hospital, Houston, TX, United States; 4 McNair Medical Institute, Baylor College of Medicine, Houston, TX, United States

**Keywords:** UPR, ER stress, insulin, processing, folding, FKBP2, ER, Golgi network

An incorrect number was provided for the **National Science Centre**. The correct number is (OPUS UMO-2020/39/B/NZ3/01408; UMO-2020/37/B/NZ3/01917).

The original article has been updated.

