# Supplementary material for: Molecular puzzle of insulin: structural assembly pathways and their role in diabetes
Source: Front Cell Dev Biol. 2025 Feb 20;13:1502469. doi: 10.3389/fcell.2025.1502469 (PMC11882602; doi:10.3389/fcell.2025.1502469)
Supplement: Supplementary file 1 [file DataSheet1.pdf]

Supplemental Table S1: Genetic mutations affecting insulin folding and ER stress.

Gene (human or mouse)	Location of a mutation (at the protein level)	Type of Genetic Change	Effect seen in the ER/on Insulin Folding	Phenotype	Species	Reference
INS	R55C	Point mutation	Disrupted proinsulin folding, retention in ER	MODY diabetes	Human	(Meur et al., 2010)
INS	G32S	Point mutation	Improper folding, aggregation in ER	Neonatal diabetes	Human	(Stoy et al., 2007)
INS	F48C	Point mutation	Disrupted disulfide bond formation, retention in ER	Neonatal diabetes	Human	(Stoy et al., 2007)
INS	R89C	Point mutation	Improper folding, ER stress	Neonatal diabetes	Human	(Stoy et al., 2007)
INS	L30M	Point mutation	Proinsulin aggregation in ER	Neonatal diabetes	Human	(Stoy et al., 2007)
INS	Y108C	Point mutation	Disrupted folding, retention in ER	Neonatal diabetes	Human	(FitzHugh and Schiller, 2023)
WFS1	-	Genetic polymorphism	Calcium dysregulation, ER stress	Association with type 2 diabetes	Human	(Sparso et al., 2008)
HSPA5	-	Polymorphism	Impaired protein folding capacity in ER	ER stress	Human	(Klyosova et al., 2024)
EIF2AK3	-	Point mutation	Dysfunction in ER stress response	Diabetes linked to *PERK* mutations	Human	(Fatani, 2019)
DNAJC3	-	Deletion/Point mutation	ER dysfunction, reduced functionality	Juvenile-onset onset diabetes	Human	(Alwatban et al., 2021;Lytrivi et al., 2021;Syno

						fzik et al., 2014)
Xbp1	-	Point mutation	Improper ER stress response	Diabetes	Mouse	(Lee et al., 2011)
Manf	-	Deletion	ER stress	Diabetes	Mouse	(Lindahl et al., 2014)
Ins2	C96Y	Point mutation	Proinsulin retention in the ER, ER stress	Diabetes (Akita model)	Mouse	(Undi et al., 2019)
Pdia1	-	Deletion	Disruptions in disulfide bond formation	Increased ER stress	Mouse	(Jang et al., 2019)

References:

Alwatban, S., Alfaraidi, H., Alosaimi, A., Alluhaydan, I., Alfadhel, M., Polak, M., et al. (2021). Case Report: Homozygous DNAJC3 Mutation Causes Monogenic Diabetes Mellitus Associated With Pancreatic Atrophy. *Front Endocrinol (Lausanne)* 12**,** 742278. doi:10.3389/fendo.2021.742278

Fatani, T.H. (2019). EIF2AK3 novel mutation in a child with early-onset diabetes mellitus, a case report. *BMC Pediatr* 19**,** 85. doi:10.1186/s12887-019-1432-8

Fitzhugh, Z.T., and Schiller, M.R. (2023). Systematic Assessment of Protein C-Termini Mutated in Human Disorders. *Biomolecules* 13, doi:10.3390/biom13020355

Jang, I., Pottekat, A., Poothong, J., Yong, J., Lagunas-Acosta, J., Charbono, A., et al. (2019). PDIA1/P4HB is required for efficient proinsulin maturation and ss cell health in response to diet induced obesity. *Elife* 8, doi:10.7554/eLife.44528

Klyosova, E.Y., Azarova, Y.E., Ilyina, E.A., Goryainova, N.V., and Polonikov, A.V. (2024). Association between Polymorphisms of Heat Shock Protein HSPA5 and Risk of Type 2 Diabetes Mellitus. *Bull Exp Biol Med* 176**,** 599-602. doi:10.1007/s10517-024-06075-2

Lee, A.H., Heidtman, K., Hotamisligil, G.S., and Glimcher, L.H. (2011). Dual and opposing roles of the unfolded protein response regulated by IRE1alpha and XBP1 in proinsulin processing and insulin secretion. *Proc Natl Acad Sci U S A* 108**,** 8885-8890. doi:10.1073/pnas.1105564108

Lindahl, M., Danilova, T., Palm, E., Lindholm, P., Voikar, V., Hakonen, E., et al. (2014). MANF is indispensable for the proliferation and survival of pancreatic beta cells. *Cell Rep* 7**,** 366-375. doi:10.1016/j.celrep.2014.03.023

Lytrivi, M., Senee, V., Salpea, P., Fantuzzi, F., Philippi, A., Abdulkarim, B., et al. (2021). DNAJC3 deficiency induces beta-cell mitochondrial apoptosis and causes syndromic young-onset diabetes. *Eur J Endocrinol* 184**,** 455-468. doi:10.1530/EJE-20-0636

Meur, G., Simon, A., Harun, N., Virally, M., Dechaume, A., Bonnefond, A., et al. (2010). Insulin gene mutations resulting in early-onset diabetes: marked differences in clinical presentation, metabolic status, and pathogenic effect through endoplasmic reticulum retention. *Diabetes* 59**,** 653-661. doi:10.2337/db09-1091

Sparso, T., Andersen, G., Albrechtsen, A., Jorgensen, T., Borch-Johnsen, K., Sandbaek, A., et al. (2008). Impact of polymorphisms in WFS1 on prediabetic phenotypes in a population-based sample of middle-aged people with normal and abnormal glucose regulation. *Diabetologia* 51**,** 1646-1652. doi:10.1007/s00125-008-1064-2

Stoy, J., Edghill, E.L., Flanagan, S.E., Ye, H., Paz, V.P., Pluzhnikov, A., et al. (2007). Insulin gene mutations as a cause of permanent neonatal diabetes. *Proc Natl Acad Sci U S A* 104**,** 15040-15044. doi:10.1073/pnas.0707291104

Synofzik, M., Haack, T.B., Kopajtich, R., Gorza, M., Rapaport, D., Greiner, M., et al. (2014). Absence of BiP co-chaperone DNAJC3 causes diabetes mellitus and multisystemic neurodegeneration. *Am J Hum Genet* 95**,** 689-697. doi:10.1016/j.ajhg.2014.10.013

Undi, R., Lim, H.Y., and Wang, W. (2019). Rapid and reliable identification of insulin 2 gene mutation in Akita diabetic mice by a tetra-primer-ARMS-PCR method. *Heliyon* 5**,** e01112. doi:10.1016/j.heliyon.2018.e01112

Supplemental Table 2. Table showing key proteins involved in insulin processing.

Participating Protein	Function in insulin processing and maturation	Reference
	Signal peptide cleavage	
**<u>Signal Peptidase complex</u>**<br>Signal peptidase complex subunit 1 / Signal peptidase complex subunit 2 / Signal peptidase complex subunit 3 / Signal peptidase complex catalytic subunit SEC11A / Signal peptidase complex catalytic subunit SEC11C<br><br>[SPCS1:Q9Y6A9, SPCS2:E9PRB9, SPCS3:P61009, SEC11A:P67812, SEC11C:Q9BY50]	Removes the signal peptide from preproinsulin in the ER as part of the signal peptidase complex, composed of subunits including SPCS1, SPCS2, SPCS3, and SEC11A-C	(Liaci et al., 2021)
	Entrance to the ER	
**<u>Protein transport protein SEC61 complex</u>**<br>Protein transport protein Sec61 subunit alpha isoform 1 / Protein transport protein Sec61 subunit alpha isoform 2<br>[SEC61A1:P61619, SEC61A2:Q9H9S3]	Forms part of the SEC61 translocon complex, creating a channel through the endoplasmic reticulum membrane for the translocation of nascent polypeptides. Functions in concert with SEC62, SEC63, and SPR	(Jadhav et al., 2015)<br>(Lee et al., 2021)<br>(Sicking et al., 2021)
**<u>Translocation protein SEC62</u>**<br>SEC62:Q99442	Assists the Sec61 complex in initiating protein translocation and maintains nascent polypeptide stability during their transfer into the ER	

**<u>Translocation protein SEC63</u>**<br>Translocation protein SEC63 homolog<br>SEC63:Q9UGP8	Cooperates with BiP to support the folding of proteins after their translocation to the ER	
**<u>Signal recognition particle complex</u>**<br>SRP complex, e.g. SRP54:P61011	Recognizes signal peptide and directs the protein-ribosome complexes to SRP receptor on the ER membrane	
	Folding in ER	
**<u>Binding immunoglobulin protein</u>**<br>Endoplasmic reticulum chaperone BiP<br>HSPA5:P11021	Protein chaperone that assists in the folding of proinsulin in the ER	(Evensen et al., 2013)<br>(Oka et al., 2013)<br>(Pobre et al., 2019)
**<u>Heat shock protein 70 family</u>**<br>[Heat shock 70 kDa protein 1A / Heat shock 70 kDa protein 1B]<br>[HSPA1A:P0DMV8, HSPA1B:P0DMV9]	Protects nascent polypeptides during translation and translocation to the ER	(Craig, 2018)
**<u>Protein disulfide isomerase (PDI)</u>**<br>P4HB:P07237	Facilitates the formation and isomerization of disulfide bonds in proinsulin	(Rajpal et al., 2012)

**Calnexin**<br>CANX:P27824	Binds to glycosylated proinsulin, promoting its stability and facilitating proper folding	(Paskevicius et al., 2023)
**Calreticulin**<br>CARL:P27797	Like calnexin, aids in the folding of glycosylated proinsulin	(Ikezaki et al., 2020)
**Glucose-Regulated Protein 94, GRP94**<br>Endoplasmin<br>HSP90B1:P14625	A protein chaperone that helps fold proteins in the ER	(Hoefner et al., 2023)<br>(Kim et al., 2024)
**FK506 Binding Protein 2**<br>Peptidyl-prolyl cis-trans isomerase FKBP2<br>FKBP2:P26885	Participates in the folding and assembly of proinsulin, accelerating the isomerization of peptidyl-prolyl bonds	(Hoefner et al., 2023)
**Endoplasmatic reticulum oxireductin 1**<br>ERO1-like protein alpha<br>ERO1:Q96HE7	Reduces oxidized PDI, thereby enabling continued disulfide bond formation	(Moilanen et al., 2018)

**<u>Heat Shock Protein 90</u>**<br>Heat shock protein HSP 90-alpha<br>HSP90AA1:P07900	This chaperone protein facilitates the folding and maturation of nascent proteins, such as insulin, by collaborating with other chaperone networks	(Kudze et al., 2018)
**<u>Peroxiredoxin-4</u>**<br>PRDX4:Q13162	Catalyzes the oxidation of PDI family proteins within the ER	(Elko et al., 2021)
**<u>Protein disulfide-isomerase A3</u>**<br>PDIA3:P30101	Protein folding chaperone that catalyzes the formation, isomerization, and redox modification of disulfide bonds	(Peaper et al., 2005)
**<u>Glutathione peroxidase 7</u>**<br>GPX7:Q96SL4	Mitigates oxidative stress in the ER by reducing hydrogen peroxide and lipid hydroperoxides, thereby contributing to proper protein folding	(Konno et al., 2021)<br>(Mehmeti et al., 2017)
**<u>Glutathione peroxidase 8</u>**<br>Probable glutathione peroxidase 8<br>GPX8:Q8TED1	Like GPX7 detoxifies peroxides in the ER, supports the maintenance of redox balance and protein quality control	
Transport to Golgi apparatus		

**Coat Protein Complex II, COPII**	Facilitates protein transport from the ER to the Golgi apparatus by forming and mediating the trafficking of transport vesicles	(Bi et al., 2007)<br>(Zanetti et al., 2011)<br>(Zanetti et al., 2013)
**COPII components (small GTP-ases)**<br>[Small COPII coat GTPase SAR1A / Small COPII coat GTPase SAR1B]<br>[SAR1A:Q9NR31, SAR1B:Q9Y6B6]	Initiate the formation of COPII vesicles. Upon GTP binding and activation, the vesicles integrate into the endoplasmic reticulum membrane, where other components of the COPII coat protein complex are recruited.	
**COPII components (SEC23/SEC24 proteins)**<br>[Protein transport protein Sec23A / Protein transport protein Sec23B / Protein transport protein Sec24A / Protein transport protein Sec24B]<br>[SEC23A:Q15436, SEC23B:Q15437, SEC24A:O95486, SEC24B:O95487]	COPII subunits that help protein recruitment to vesicles	
**COPII components (SEC13/SEC31 proteins)**<br>[Protein SEC13 homolog / Protein transport protein Sec31A]<br>[SEC13:P55735, SEC31A:O94979]	The components form the outer coat of the COPII vesicle	
**COPII components (vesicular transport proteins, examples)**<br>[General vesicular transport factor p115 / Ras-related protein Rab-1A / Golgi SNAP receptor complex member 1]<br>**[P115:O60763, RAB1A:P62820, GOSR1:F6RU00]**	The components participate in the process of vesicle transport between the ER and the Golgi apparatus, which is crucial for the proper transport of proteins and maintaining the cell function.	(D'Souza et al., 2021)<br>(Bravo-Plaza et al., 2023)<br>(Gmachl and Wimmer, 2001)

Processing in the Golgi Apparatus		
**<u>Prohormone convertase 1/3 (PC1/3)</u>**<br>Neuroendocrine convertase 1<br>PCSK1:P29120	Converts proinsulin into mature insulin and C-peptide.<br>PC1/3 cleaves COOH-terminal to proinsulin Arg31-Arg32 (B-chain/C-peptide junction) of proinsulin. Note: Arg31-Arg32 refers to Arg55-Arg56 in preproinsulin, using canonical human INS sequence P01308-1.	(Germanos et al., 2021)<br>(Omar-Hmeadi and Idevall-Hagren, 2021)
**<u>Prohormone convertase 2 (PC2)</u>**<br>**Prohormone convertase 2**<br>PC2:Q9UM69	Cooperates with PC1/3 in the processing of proinsulin.<br>Preferentially cleaves at the Lys64-Arg65 site (C-peptide/A-chain junction) of proinsulin. Note: Lys64-Arg65 refers to Lys88-Arg89 in preproinsulin, using canonical human INS sequence P01308-1.	
**<u>Carboxypeptidase E (CPE)</u>**<br>CPE:P16870	Finalizes the conversion of proinsulin to mature insulin	
**<u>Mannosidase I (enzyme family)</u>**<br>[Mannosyl-oligosaccharide 1,2-alpha-mannosidase IA / Mannosyl-oligosaccharide 1,2-alpha-mannosidase IB / Mannosyl-oligosaccharide 1,2-alpha-mannosidase IC]<br>[MAN1A1:P33908, MAN1A2:O60476, MAN1C1:Q9NR34]	Catalyze the removal of mannose residues from proteins. Involved in the maturation of Asn-linked oligosaccharides	(Liebminger et al., 2009)
**<u>N-acetylglucosaminyltransferase</u>**<br>Beta-1,4-mannosyl-glycoprotein 4-beta-N-acetylglucosaminyltransferase<br>MGAT3:Q09327	Adds N-acetylglucosamine moiety to proteins	(Pinho et al., 2009)

Supplemental Table 2. Table showing key proteins involved in insulin processing. The table is divided into stages occurring during insulin processing: signal peptide cleavage, entrance to the endoplasmic reticulum (ER), folding in ER, transport to the Golgi apparatus, and processing in the Golgi. The unique identifiers refer to protein names and gene symbols with the reference to human UniProtKB/Swiss-Prot reviewed dataset.

References

Bi, X., Mancias, J. D. & Goldberg, J. (2007). Insights into COPII coat nucleation from the structure of Sec23.Sar1 complexed with the active fragment of Sec31. *Dev Cell*, 13**,** 635-645. https://doi.org/10.1016/j.devcel.2007.10.006.

Bravo-Plaza, I., Tagua, V. G., Arst, H. N., Alonso, A., Pinar, M., Monterroso, B., et al. (2023). The Uso1 globular head interacts with SNAREs to maintain viability even in the absence of the coiled-coil domain. *Elife*, 12. https://doi.org/10.7554/eLife.85079.

Craig, E. A. (2018). Hsp70 at the membrane: driving protein translocation. *BMC Biol*, 16**,** 11. https://doi.org/10.1186/s12915-017-0474-3.

D'Souza, Z., Sumya, F. T., Khakurel, A. & Lupashin, V. (2021). Getting Sugar Coating Right! The Role of the Golgi Trafficking Machinery in Glycosylation. *Cells*, 10. https://doi.org/10.3390/cells10123275.

Elko, E. A., Manuel, A. M., White, S., Zito, E., van der Vliet, A., Anathy, V. & Janssen-Heininger, Y. M. W. (2021). Oxidation of peroxiredoxin-4 induces oligomerization and promotes interaction with proteins governing protein folding and endoplasmic reticulum stress. *J Biol Chem*, 296**,** 100665. https://doi.org/10.1016/j.jbc.2021.100665.

Evensen, N. A., Kuscu, C., Nguyen, H. L., Zarrabi, K., Dufour, A., Kadam, P., et al. (2013). Unraveling the role of KIAA1199, a novel endoplasmic reticulum protein, in cancer cell migration. *J Natl Cancer Inst*, 105**,** 1402-16. https://doi.org/10.1093/jnci/djt224.

Germanos, M., Gao, A., Taper, M., Yau, B. & Kebede, M. A. (2021). Inside the Insulin Secretory Granule. *Metabolites*, 11. https://doi.org/10.3390/metabo11080515.

Gmachl, M. J. & Wimmer, C. (2001). Sequential involvement of p115, SNAREs, and Rab proteins in intra-Golgi protein transport. *J Biol Chem*, 276**,** 18178-84. https://doi.org/10.1074/jbc.M101513200.

Hoefner, C., Bryde, T. H., Pihl, C., Tiedemann, S. N., Bresson, S. E., Hotiana, H. A., et al. (2023). FK506-Binding Protein 2 Participates in Proinsulin Folding. *Biomolecules*, 13. https://doi.org/10.3390/biom13010152.

Ikezaki, M., Minakata, S., Nishitsuji, K., Tabata, S., Lee Matsui, I. S., Takatani, M., et al. (2020). Calreticulin protects insulin against reductive stress in vitro and in MIN6 cells. *Biochimie*, 171-172**,** 1-11. https://doi.org/10.1016/j.biochi.2020.01.011.

Jadhav, B., McKenna, M., Johnson, N., High, S., Sinning, I. & Pool, M. R. (2015). Mammalian SRP receptor switches the Sec61 translocase from Sec62 to SRP-dependent translocation. *Nat Commun*, 6**,** 10133. https://doi.org/10.1038/ncomms10133.

Kim, D. S., Song, L., Gou, W., Kim, J., Liu, B., Wei, H., et al. (2024). GRP94 is an IGF-1R chaperone and regulates beta cell death in diabetes. *Cell Death Dis*, 15**,** 374. https://doi.org/10.1038/s41419-024-06754-y.

Konno, T., Melo, E. P., Chambers, J. E. & Avezov, E. (2021). Intracellular Sources of ROS/H(2)O(2) in Health and Neurodegeneration: Spotlight on Endoplasmic Reticulum. *Cells*, 10. https://doi.org/10.3390/cells10020233.

Kudze, T., Mendez-Dorantes, C., Jalloh, C. S. & McClellan, A. J. (2018). Evidence for interaction between Hsp90 and the ER membrane complex. *Cell Stress Chaperones*, 23**,** 1101-1115. https://doi.org/10.1007/s12192-018-0908-z.

Lee, J. H., Jomaa, A., Chung, S., Hwang Fu, Y. H., Qian, R., Sun, X., et al. (2021). Receptor compaction and GTPase rearrangement drive SRP-mediated cotranslational protein translocation into the ER. *Sci Adv*, 7. https://doi.org/10.1126/sciadv.abg0942.

Liaci, A. M., Steigenberger, B., Telles de Souza, P. C., Tamara, S., Grollers-Mulderij, M., Ogrissek, P., et al. (2021). Structure of the human signal peptidase complex reveals the determinants for signal peptide cleavage. *Mol Cell*, 81**,** 3934-3948 e11. https://doi.org/10.1016/j.molcel.2021.07.031.

Liebminger, E., Huttner, S., Vavra, U., Fischl, R., Schoberer, J., Grass, J., et al. (2009). Class I alpha-mannosidases are required for N-glycan processing and root development in Arabidopsis thaliana. *Plant Cell*, 21**,** 3850-67. https://doi.org/10.1105/tpc.109.072363.

Mehmeti, I., Lortz, S., Avezov, E., Jorns, A. & Lenzen, S. (2017). ER-resident antioxidative GPx7 and GPx8 enzyme isoforms protect insulin-secreting INS-1E beta-cells against lipotoxicity by improving the ER antioxidative capacity. *Free Radic Biol Med*, 112**,** 121-130. https://doi.org/10.1016/j.freeradbiomed.2017.07.021.

Moilanen, A., Korhonen, K., Saaranen, M. J. & Ruddock, L. W. (2018). Molecular analysis of human Ero1 reveals novel regulatory mechanisms for oxidative protein folding. *Life Sci Alliance*, 1**,** e201800090. https://doi.org/10.26508/lsa.201800090.

Oka, O. B., Pringle, M. A., Schopp, I. M., Braakman, I. & Bulleid, N. J. (2013). ERdj5 is the ER reductase that catalyzes the removal of non-native disulfides and correct folding of the LDL receptor. *Mol Cell*, 50**,** 793-804. https://doi.org/10.1016/j.molcel.2013.05.014.

Omar-Hmeadi, M. & Idevall-Hagren, O. (2021). Insulin granule biogenesis and exocytosis. *Cell Mol Life Sci*, 78**,** 1957-1970. https://doi.org/10.1007/s00018-020-03688-4.

Paskevicius, T., Farraj, R. A., Michalak, M. & Agellon, L. B. (2023). Calnexin, More Than Just a Molecular Chaperone. *Cells*, 12. https://doi.org/10.3390/cells12030403.

Peaper, D. R., Wearsch, P. A. & Cresswell, P. (2005). Tapasin and ERp57 form a stable disulfide-linked dimer within the MHC class I peptide-loading complex. *EMBO J*, 24**,** 3613-23. https://doi.org/10.1038/sj.emboj.7600814.

Pinho, S. S., Reis, C. A., Paredes, J., Magalhaes, A. M., Ferreira, A. C., Figueiredo, J., et al. (2009). The role of N-acetylglucosaminyltransferase III and V in the post-transcriptional modifications of E-cadherin. *Hum Mol Genet*, 18**,** 2599-608. https://doi.org/10.1093/hmg/ddp194.

Pobre, K. F. R., Poet, G. J. & Hendershot, L. M. (2019). The endoplasmic reticulum (ER) chaperone BiP is a master regulator of ER functions: Getting by with a little help from ERdj friends. *J Biol Chem*, 294**,** 2098-2108. https://doi.org/10.1074/jbc.REV118.002804.

Rajpal, G., Schuiki, I., Liu, M., Volchuk, A. & Arvan, P. (2012). Action of protein disulfide isomerase on proinsulin exit from endoplasmic reticulum of pancreatic beta-cells. *J Biol Chem*, 287**,** 43-47. https://doi.org/10.1074/jbc.C111.279927.

Sicking, M., Lang, S., Bochen, F., Roos, A., Drenth, J. P. H., Zakaria, M., et al. (2021). Complexity and Specificity of Sec61-Channelopathies: Human Diseases Affecting Gating of the Sec61 Complex. *Cells*, 10. https://doi.org/10.3390/cells10051036.

Zanetti, G., Pahuja, K. B., Studer, S., Shim, S. & Schekman, R. (2011). COPII and the regulation of protein sorting in mammals. *Nat Cell Biol*, 14**,** 20-8. https://doi.org/10.1038/ncb2390.

Zanetti, G., Prinz, S., Daum, S., Meister, A., Schekman, R., Bacia, K. & Briggs, J. A. (2013). The structure of the COPII transport-vesicle coat assembled on membranes. *Elife*, 2**,** e00951. https://doi.org/10.7554/eLife.00951.
